# Integrated Proteomics and Metabolomics Analysis of Perirenal Adipose Tissue in Obese Rabbits Treated with a Restricted Diet

**DOI:** 10.3390/biology10040321

**Published:** 2021-04-12

**Authors:** Jiahao Shao, Ting Pan, Jie Wang, Tao Tang, Yanhong Li, Xianbo Jia, Songjia Lai

**Affiliations:** 1 College of Animal Science and Technology, Sichuan Agricultural University, Chengdu 611130, China; shaojh1997@126.com (J.S.); wjie68@163.com (J.W.); m18483220592@163.com (T.T.); lyh81236718@163.com (Y.L.); jaxb369@sicau.edu.cn (X.J.); 2 College of Veterinary Medicine, Sichuan Agricultural University, Chengdu 611130, China; panting555666@163.com

**Keywords:** proteomics, metabolomics, rabbits, dieting

## Abstract

**Simple Summary:**

Nowadays, obesity and obesity-related diseases are rapidly increasing in most countries and regions. In this context, about 25-50% of people show that they are trying to lose weight and this trend is especially prominent among women. Here, obese rabbits were used as a model to study the effects of dieting on the molecular change of perirenal adipose tissue by integrating proteomics and metabolomics analysis. Our results indicate that 343 proteins and 150 metabolites were markedly changed and these molecules are associated with amino acid metabolism, lipid metabolism, and membrane and cytoskeleton reconstruction. Interestingly, some inflammation-related molecules such as mevalonic acid, arachidonic acid, 15(S)-HpETE, cholecalciferol, hydrocortisone, lipoxin B4, lithocholic acid, etc. were differently changed and these molecules may be the key roles to fight inflammation induced by a high-fat diet. In conclusion, this study provides a comprehensive overview of the molecular profile of dieting-mediated weight loss and may provide some help for the prevention and treatment of obesity.

**Abstract:**

In recent years, many people have shown an excess of fat accumulation. Known as obesity, this lesion poses an increased risk for multiple diseases, such as endocrine disease, diabetes, and cancer, and has reached epidemic proportions. Accompanied by the development of obesity, concern over body image and weight loss behavior is a growing social problem and public health threat, causing concern for many health professionals. However, the consequences of rapid weight loss remain largely unclear. Here, we applied an integrated proteomics and metabolomics analysis to investigate the effects of dieting on the proteins and metabolites in obese rabbits. Our study revealed that 343 differentially expressed proteins (136 upregulated and 207 downregulated) and 150 differentially expressed metabolites (91 upregulated and 59 downregulated) were identified. These molecules are mainly involved in the biological processes, including amino acid metabolism, lipid metabolism, and membrane and cytoskeleton reconstruction. The integrated analysis found that mevalonic acid, arachidonic acid, 15(S)-HpETE, cholecalciferol, hydrocortisone, lipoxin B4, lithocholic acid, etc. were associated with multiple pathways, and they may be the key factors to fight inflammation induced by a high-fat diet (HFD). Overall, this study provides further insight into the consequences of dieting-mediated weight loss and may contribute to the prevention and treatment of obesity.

## 1. Introduction

Nowadays, obesity promotes an increased risk for a series of diseases, including endocrine disease, diabetes, cancer, etc., and its incidence rapidly increases in most countries and even in some lower-income countries [1]. Obesity is visualized as an excess of adipose tissue, which is an endocrine organ and energy storage organ and plays a key role in maintaining physiological activities [2]. Obesity is a complex pathological process, and available and cheaper highly palatable and fat-dense foods are important contributors [3,4]. In this context, young females are suffering from a higher rate of obesity risk than males [5]. Meanwhile, concern over body image and weight loss behavior is particularly common in adolescent females [6]. Overall, slimness is often considered the norm for physical appearance. Dieting seems to be the first choice for obese people to lose weight because of its easy applicability. Despite that, there are some controversies about dieting to lose weight, such as the measurement of dieting and psychological reactions for different people [7,8]. The harmful side effects of the thinness pursuit are a growing social problem and public health threat, causing concern for many health professionals, and the consequences of dieting need to be taken seriously [9].

Omics technology is developing at a high rate of speed and is broadly applied as a powerful tool for various studies, such as the examination of pathophysiological processes, identification of biomarkers, molecular profiling, and characterization of complex biochemical systems [10]. The physiological activity is complex, as using a single omics makes it hard to fully reveal the potential mechanism. Thus, to identify the potential biological molecules and improve the understanding of the overall biological mechanisms, an integrated analysis of multiple omics is necessary. Moreover, different omics are complementary to each other and contribute to increasing the reliability of the results. Among these integrated omics, an integrated proteomics and metabolomics analysis is a useful combination to reveal the mechanisms of molecular regulation on phenotypic changes. To our knowledge, no combination of proteomics and metabolomics has been performed to analyze the molecular changes of perirenal adipose tissue from obese people or animals treated with dieting. Thus, we aimed to gain further understanding of the molecular mechanisms of losing weight through an integrated proteomics and metabolomics analysis from the obese rabbit model under dietary restriction treatment.

## 2. Materials and Methods

### 2.1. Animals

The animals were sixteen female Tianfu black rabbits (a new variety screened by Sichuan Agricultural University) that were 35 days old at the initiation of this trial. Rabbits were kept in individual cages (600 mm × 600 mm × 500 mm) and were placed indoors (21 to 23 °C, 60% humidity). Moreover, rabbits were treated with a two-stage diet plan. In the first stage, rabbits were fed a high-fat diet (HFD; a 10% lard was added to the standard diet, 120 g/d) for 35 days. In the second stage, HFD rabbits were treated with a restricted diet (RD; standard diet, 20 g/d) for 20 days. Before each stage, rabbits were housed undisturbed for 7 days to adapt to the change. Animals were classified as obese using the method described in our previous study [11]. Briefly, body weight, body length, bust length, and adipose tissue weight were used as the markers of obesity. Lastly, unqualified (weak, disabled, and sick) rabbits were eliminated, and six rabbits from the HFD (77 days) and RD (104 days) groups were selected for sampling. All experimental protocols were performed under the direction of the Institutional Animal Care and Use Committee from the College of Animal Science and Technology, Sichuan Agricultural University, China (DKY-B2019202015).

### 2.2. Histomorphological Observation

To examine the histomorphological changes of perirenal adipose tissue, selective rabbits were sacrificed (electric shock and then exsanguination) for sampling, and perirenal adipose tissues were stained with hematoxylin–eosin (HE). Briefly, tissue samples were fixed with a 10% neutral formaldehyde fixator for 24 h and then washed with water. The tissue specimens were successively dehydrated and embedded in paraffin, followed by HE staining. Finally, about 5-μm-thick sections were obtained using a microtome (RM2235, Leica, Nussloch, Germany). Moreover, an optical microscope (DM1000, Leica, Nussloch, Germany) was used to capture images at a 200× field of view, and the relevant parameters (adipocytes area, diameter, number, and density) were measured by image-pro plus 6.0 software (Media Cybernetics, Inc, Rockville, MD, USA).

### 2.3. TMT Labeling

Total protein was extracted and measured using a commercial kit (Sangon, Shanghai, China) and the BCA Protein Assay Kit (Beyotime, Jiangsu, China) following the manufacturer’s protocols, respectively. Only qualified protein (concentration > 1 mg/mL) was used for further trial. One hundred and twenty micrograms of protein were diluted with a dissolution buffer (1.5-μg trypsin and 500 μL of 100-mM triethylammonium bicarbonate (TEAB) buffer to 100 μL) and digested at 37 °C. Subsequently, 1.5-μg trypsin and CaCl_2_ were added to the sample. The next day, formic acid was mixed with the above-digested sample (adjusted pH under 3), and the mixture was centrifuged at 12,000× *g* for 5 min at an indoor temperature. The supernatant was loaded onto an C18 desalting column (Phenomenex, Torrance, CA, USA), washed with washing buffer (0.1% formic acid and 3% acetonitrile) 3 times, then eluted by elution buffer (0.1% formic acid and 70% acetonitrile). The eluent was collected and lyophilized. One hundred microliters of 0.1-M TEAB buffer and 41 μL of acetonitrile-dissolved tandem mass tag (TMT) reagent were added and incubated for 2 h. The mixture was stopped by adding 8% ammonia. Finally, the labeling sample was desalted, pooled, and lyophilized.

### 2.4. Separation of Fractions

Mobile phases A (2% acetonitrile) and B (98% acetonitrile) were used to develop a gradient elution. The lyophilized powder was dissolved in solution A and centrifuged at 12,000× *g* for 10 min at an indoor temperature. The sample was fractionated using a C18 column (4.6-mm ID, 250-mm length, and 5-μm particles) on a Rigol L3000 high performance liquid chromatography (HPLC) system, and the column oven was set as 50 °C. The eluates were monitored at UV 214 nm, collected for a tube per minute, and combined into 10 fractions. All fractions were dried under vacuum and then reconstituted in 0.1% (*v*/*v*) formic acid (FA) in water.

### 2.5. LC-MS/MS Analysis

The tryptic peptides were detached by using the EASY-nLC 1200 ultra performance liquid chromatography (UHPLC) system (Thermo Fisher Scientific, Waltham, MA, USA). Subsequently, the separated peptides were analyzed by using a Q Exactive HF-X mass spectrometer (Thermo Fisher Scientific, Waltham, MA, USA), with an ion source of Nanospray Flex™ electron spray lonization (ESI). The ion source was set at 2.3 kV, and the capillary temperature of the ion transport was 320 °C. The m/z scan range was 350 to 1500, and the resolution was 60,000. The automatic gain control target value was 3 × 10^6^, and the maximum ion injection time was 20 ms. The top 40 precursors with the highest contents in the full scans were selected and fragmented by higher-energy collisional dissociation and analyzed by tandem mass spectrometry (MS/MS) (resolution: 45,000, automatic gain control (AGC) target value: 5 × 10^4^, maximum ion injection time: 86ms, normalized collision energy: 32%, intensity threshold: 1.2 × 10^5^, and dynamic exclusion parameter: 20 s).

### 2.6. Proteomics Data Processing and Analysis

The Proteome Discoverer 2.2 (PD; Thermo) was used to obtain the MS/MS results. The parameters were set as follows: (a) mass tolerance for a precursor ion was 10 ppm, and the mass tolerance for fragment ion was 0.02 Da, (b) carbamidomethyl was specified as a fixed modification, but the oxidation of methionine and TMT plex were specified as dynamic modifications, (c) acetylation and TMT plex were specified as N-terminal modification in PD 2.2, and (d) a maximum of 2 miscleavage sites were allowed. The protein concluding at least one unique peptide and meeting criteria (false discovery rate (FDR) ≤ 1%) was retained for further trial. The proteins with *p* < 0.05 and fold changes ≥ 1.2 or fold changes ≤ 0.83 were identified as upregulated or downregulated differentially expressed (DE) protein, respectively. Gene Ontology (GO; http://www.geneontology.org/, accessed on 16 May 2020) was used to analyze the functional annotation.

### 2.7. UHPLC-MS/MS Analysis

The detailed procedure for sample preparation was described previously [12]. Then, the metabolites were analyzed using a UHPLC system (Thermo Fisher Scientific, Waltham, MA, USA) coupled with an Orbitrap Q Exactive^TM^ HF-X mass spectrometer (Thermo Fisher Scientific, Waltham, MA, USA) by Novogene Co., Ltd. (Beijing, China). Briefly, the sample was injected into a column (2.1-mm ID, 100-mm length, and 1.9-μm particles) using a linear gradient at a flow rate of 0.2 mL/min. The eluents for the positive polarity mode were eluent A (0.1% FA in water) and eluent B (methanol) and, for the negative polarity mode, were eluent A (5-mM ammonium acetate) and eluent B (methanol). The searched parameters are set as follows: (a) spray voltage was 3.2 kV, (b) capillary temperature was 320 °C, (c) sheath gas flow was 40 arb, and aux gas flow was 10 arb.

### 2.8. Metabolomics Data Processing and Analysis

Data generated by UHPLC-MS/MS were loaded into Compound Discoverer 3.1 (CD; Thermo) to process the peak alignment, peak picking, and quantitation. Peak intensities were normalized to the total spectral intensity. The normalized data were used for a multivariate statistical analysis and were matched with the mzCloud (https://www.mzcloud.org/, accessed on 16 May 2020), mzVault, and MassList databases to obtain the accurate result. Normal transformations were attempted using the area normalization method when the data were not normally distributed. To better observe the inter-group distributions and otherness, SIMCA-*p* 11.0 software was used to carry out a partial least squares discriminant analysis (PLS-DA). Metabolites were considered to be differentially expressed, with a threshold variable importance in the projection (VIP) > 1 and fold changes > 1.5 or fold changes < 0.667 and *p* < 0.05. All DE metabolites were annotated with the Human Metabolome database (HMDB; https://hmdb.ca/metabolites, accessed on 16 May 2020) and LIPID MAPS database (https://www.lipidmaps.org/, accessed on 16 May 2020) to obtain a systematized overview of these DE metabolites. Moreover, the metabolite–metabolite interaction network helps to highlight potential functional relationships between a wide set of annotated metabolites and was built using the MetaboAnalyst database (https://www.metaboanalyst.ca/MetaboAnalyst/home.xhtml, accessed on 16 May 2020).

### 2.9. Integrated Analysis of Proteomics and Metabolomics

To further analyze the significantly changed pathways in both proteomics and metabolomics, we integrated the analysis of the DE proteins and DE metabolites in the Integrated Molecular Pathway Level Analysis (IMPaLA) database (http://impala.molgen.mpg.de/, accessed on 16 May 2020). The IMPaLA database combines information from multiple databases, such as Reactome (http://www.reactome.org, accessed on 16 May 2020), Wikipathways (https://www.wikipathways.org/index.php/WikiPathways, accessed on 16 May 2020), Kyoto Encyclopedia of Eenes and Eenomes (KEGG; https://www.kegg.jp/, accessed on 16 May 2020), and HumanCyc (https://humancyc.org/, accessed on 16 May 2020) databases.

### 2.10. Statistical Analysis

The data are expressed as the mean ± SEM. Statistical analyses were performed using the software SPSS 22.0 (Chicago, IL, USA). Volcano map and a hierarchical clustering analysis were performed using the R package. Moreover, we applied a *t*-test to calculate the statistical significance, and *p* < 0.05 was considered as statistically significant.

## 3. Results

### 3.1. Histomorphological Changes in the Perirenal Adipose Tissue of Dieting Rabbits

**Figure 1:**
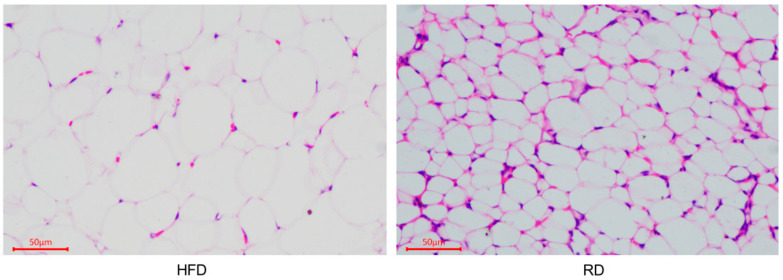
Histomorphological changes in the perirenal adipose tissue of dieting rabbits. Histomorphological image of the perirenal adipose tissue from the high-fat diet (HFD) and restricted diet (RD) rabbits.

We performed HE staining on perirenal adipose tissue from the HFD rabbits and RD rabbits to measure the histomorphological changes (Figure 1). The detailed parameters (adipocytes area, diameter, number, and density) are shown in Table 1. When comparing the significant analysis data between the HFD and RD rabbits, we found that the adipocytes area (*p* < 0.05) and adipocytes diameter (*p* < 0.01) were significantly lower in the RD rabbits, but the adipocytes number (*p* < 0.01) and adipocytes density (*p* < 0.01) in the RD rabbits were significantly higher than in the HFD rabbits. These observations indicate that dieting caused adipocytes to appear smaller in the RD group.

### 3.2. Identification and Analysis of DE Proteins

**Figure 2:**
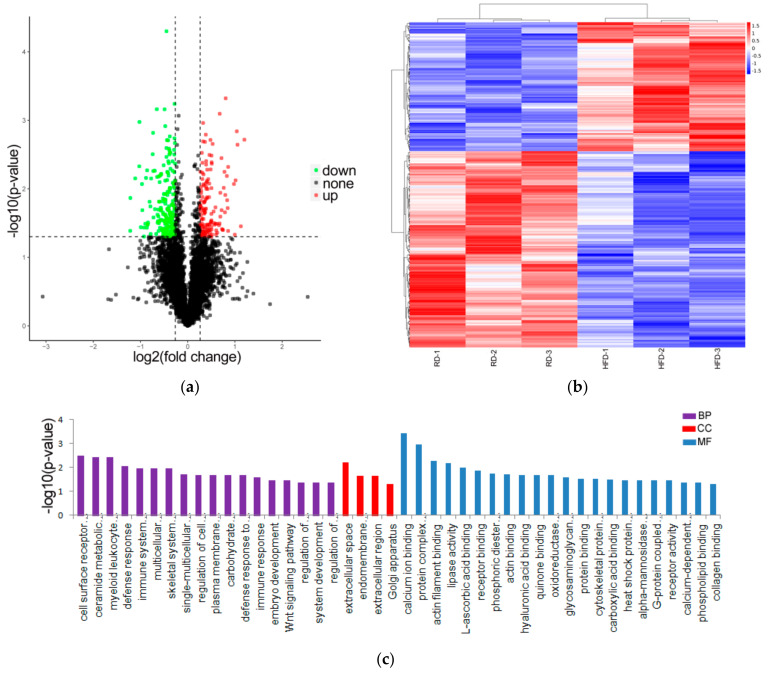
Identification and analysis of differentially expressed (DE) proteins. (**a**) Volcano map of DE proteins screened the HFD (*n* = 3) and RD (*n* = 3) groups and was built based on log2(fold change) and -log10(*p*-value). (**b**) Hierarchical clustering analysis of the DE proteins in the samples of HFD (*n* = 3) and RD (*n* = 3) (red represents upregulated proteins, and blue represents downregulated proteins). (**c**) The results from the DE proteins were applied to a GO analysis, only showing the significantly enriched GO terms (*p* < 0.05). High-fat diet: HFD and restricted diet: RD.

A total of 4922 proteins (at least one peptide and FDR ≤ 1%) were identified between the HFD and RD groups (Table S1). According to the selection criteria, of the 4922 investigated proteins, 343 proteins were classed as DE proteins in the perirenal adipose tissue between the HFD and RD groups (Table S2). Among these, 136 proteins were upregulated and 207 proteins were downregulated in the RD group compared with the HFD group (Figure 2a). DE proteins with similar expression levels across different samples were screened and grouped using a hierarchical cluster analysis (Figure 2b).

To further determine and classify the DE proteins according to their biological functions, we performed a Gene Ontology (GO) analysis with 343 DE proteins and GO terms related to DE proteins. The analysis results revealed that 342 GO terms were annotated, and 18 GO terms in biological processes (BP), 4 GO terms in cellular components (CC), and 22 GO terms in molecular functions (MF) were significantly enriched (Table S3 and Figure 2c). Among these, some GO terms were involved in the lipase activity, actin binding, phosphoric diester hydrolase activity, and actin filament binding.

### 3.3. Identification and Analysis of DE Metabolites

**Figure 3:**
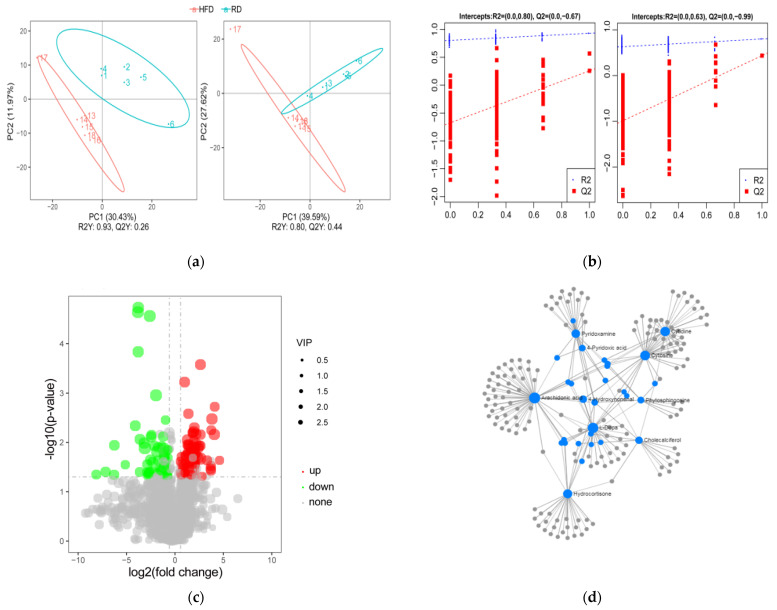
Identification and analysis of DE metabolites. (**a**) Partial least squares discriminant analysis (PLS-DA) of all metabolites in the samples of HFD (*n* = 6) and RD (*n* = 6) groups in the positive (left) and negative (right) ion modes. (**b**) The permutation test. (**c**) Volcano map of the DE metabolites screened between the HFD (*n* = 6) and RD (*n* = 6) groups and was built based on log2(fold change) and -log10(*p*-value). (**d**) The metabolite–metabolite interaction network was built by using the MetaboAnalyst database. High-fat diet: HFD and restricted diet: RD.

In our study, untargeted metabolomics based on UHPLC-MS/MS technology were used to measure the metabolite profiles of HFD and RD rabbits. The results of the PLS-DA analysis showed clear segregation between the HFD and RD groups under both positive and negative modes, indicating a faithful representation of the data and good predictability (Figure 3a). Moreover, permutation tests validated that the PLS-DA model was no overfitting of data, revealing that dieting led to significant metabolic variations between the groups (Figure 3b).

After dietary restriction treatment, a total of 150 DE metabolites were identified between the groups by the criteria of VIP > 1 and fold changes > 1.5 or fold changes < 0.667 and *p* < 0.05 (Table S4). Among these, 91 metabolites were upregulated, and 59 metabolites were downregulated (Figure 3c). When DE metabolites were compared with the Human Metabolome database (HMDB) database, 44 metabolites were annotated and classified as lipids and lipid-like molecules (*n* = 22), organic acids and derivatives (*n* = 6), organoheterocyclic compounds (*n* = 5), etc. (Table S5). Moreover, metabolites annotated with the LIPID MAPS database were mainly classified as fatty acyls (FTA; *n* = 10), glycerophospholipids (GP; *n* = 13), polyketides (PK; *n* = 2), and sterol lipids (ST; *n* = 6) (Table S6). The metabolite–metabolite interactions network showed that arachidonic acid (AA), L-dopa, cholecalciferol, hydrocortisone, and pyridoxamine are at the center of the network, indicating that these metabolites may play an important role in the process of dieting (Figure 3d).

### 3.4. Integrated Analysis of Proteomics and Metabolomics

**Figure 4:**
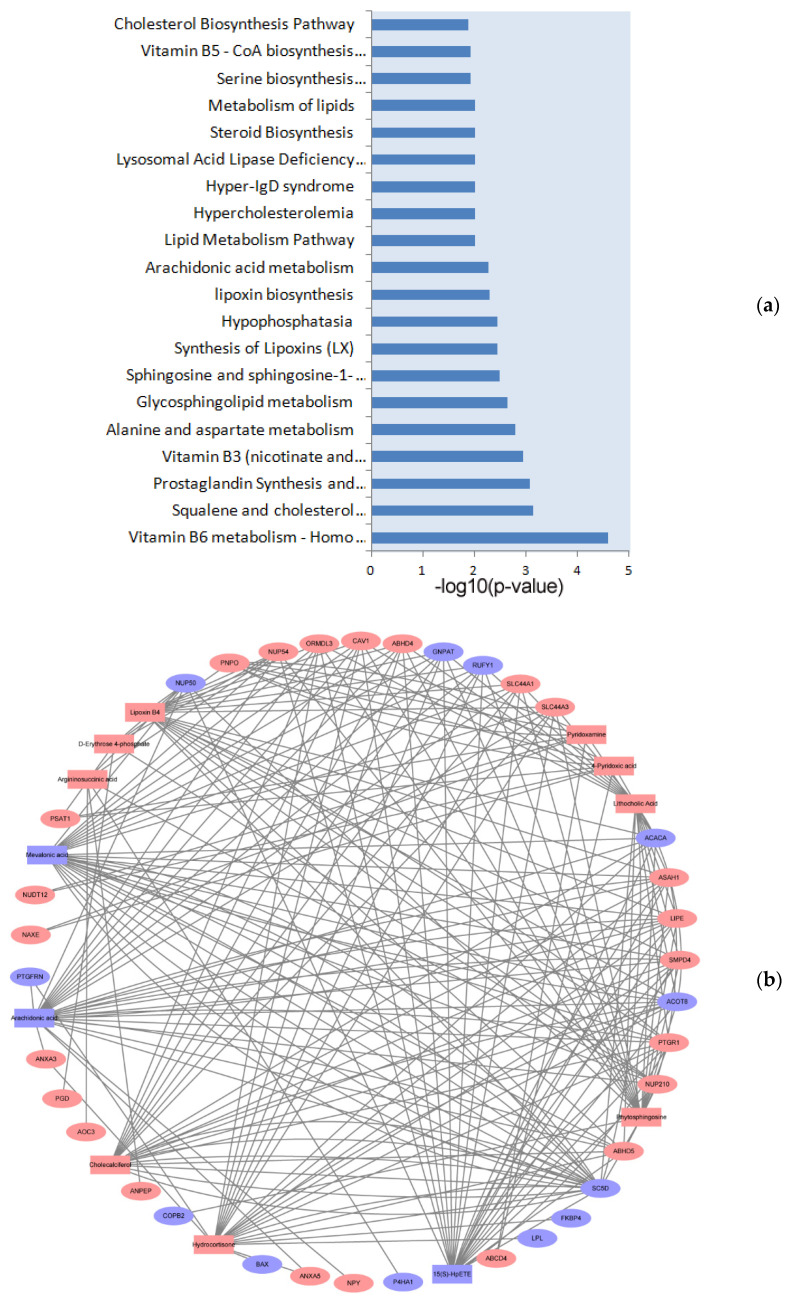
Integrated analysis of proteomics and metabolomics. (**a**) The integrated pathways analysis results of proteomics and metabolomics, only showing the top 20 pathways (*p* < 0.05). (**b**) We chose the pathways as an intermediate carrier, and the proteins and metabolites network were obtained. The circle represents proteins, and the triangle represents metabolites. Red represents the upregulated molecule, and blue represents the downregulated molecule. The line number indicates that proteins and metabolites are involved in multiple pathways. High-fat diet: HFD and restricted diet: RD.

To further identify the metabolic pathways impacted by dieting in obese rabbits, the MetaboAnalyst database was used to perform an integrative pathway analysis of the proteomics and metabolomics. According to the analysis results, some key pathways were considered to be associated with lipid metabolism, energy metabolism, vitamin metabolism, hormone metabolism, and amino acid metabolism and were significantly enriched (*p* < 0.05) after the dieting treatment, such as vitamin B6 metabolism, steroids metabolism, metabolism of lipids, and cholesterol metabolism, and fat digestion and absorption and free fatty acids regulate insulin secretion, etc. (Table S7 and Figure 4a). To better understand the changed pathways, the relationship network between the DE proteins and DE metabolites involved in significant pathways is illustrated in Figure 4b. Among these, mevalonic acid (MVA), AA, cholecalciferol, etc. were associated with multiple proteins, indicating that their interactions may play an important role in the regulation of dieting in obese rabbits.

## 4. Discussion

Nowadays, the rapid epidemic of obesity poses a serious challenge to the prevention and treatment of chronic diseases throughout the world, including cardiovascular, diabetes, and some cancers [13]. The obesity epidemic has reached epidemic proportions not only in developed but, also, in developing countries. Thus, choosing a reasonable strategy to lose weight is important in tackling increasing obesity trends [14]. In our study, we established an obese rabbit model to investigate the molecular alterations underlying dieting by using an integrated proteomics and metabolomics analysis. However, only a limited number of proteins, metabolites, and pathways were discussed due to space limitations.

White adipose tissue (WAT) is a multi-depot organ that plays a regulatory role in energy homeostasis by adjusting the triglyceride (TG) storage [15]. Lipolysis and lipogenesis are key processes determining mature adipocyte sizes and mass. Here, when comparing the histomorphology between the HFD and RD rabbits, we found that RD caused adipocytes to appear smaller, indicating that lipolysis was increased or lipogenesis was decreased. In fact, the hydrolysis of fatty acids from TG stored in cellular lipid droplets is essential to ensure an adequate energy supply [16]. TG is gradually hydrolyzed by multifold lipases to fatty acids and glycerol and further to release into the blood for biological oxidation by multiple organs. In this process, adipocyte hormonal-sensitive lipase (HSL) is activated, and the TG stored in cellular lipid droplets is hydrolyzed, and the adipocyte volume is reduced [17]. Moreover, lipoprotein lipase (LPL) is also considered to be a key enzyme for lipid and lipoproteins metabolism [18]. The emerging data suggest the relative levels of LPL activity in adipose tissue determine whether lipids are partitioned towards storage or utilization and thereby lead to weight gain or loss [19]. During dieting, the LPL activity is decreased in adipose tissue and associated with an increase in the activity of the lipase [19,20]. HSL is known to hydrolyze TG, diglycerides (DG), cholesteryl esters, and retinyl esters, thereby providing the body with an energy substrate [21]. Such reciprocal regulation has probably evolved to maximize energy storage during periods of food availability and energy availability when food is scarce [19]. Acetyl-CoA carboxylase alpha (ACACA) is rate-limiting for the synthesis of long-chain fatty acids [22]. Our study showed that ACACA was lower in the RD group, suggesting that the body maintains an energy supply during starvation by hydrolyzing fat and inhibiting its synthesis. Moreover, the proteomic analysis also indicated an upregulation of the sphingolipid metabolism pathway (N-acylsphingosine amidohydrolase 1 and sphingomyelin phosphodiesterase 4) and adipocytokine signaling pathway (CD36 molecule, neuropeptide Y, and phosphoenolpyruvate carboxykinase 1) in the RD group. Sphingolipid biosynthesis is required for adipocyte cell viability and normal metabolic function [23]. The dysregulation of the adipocytokine signaling pathway is related to eating disorders and may be the common pathway for body weight regulation in complex diseases related to unhealthy lifestyles [24]. Together, these findings suggest that RD leads to a decrease in adipocyte sizes and a series of disorders in the lipid metabolism in female obese rabbits. However, whether resuming a normal or HFD after dieting will restore obesity needs further study.

Lipolysis is a dynamic process involving an elaborate network of allosteric regulators, hormones, signaling pathways, and transcription factors, etc. Among these 343 DE proteins, some proteins were reported to be associated with amino acid metabolism, suggesting the dysregulation of amino acid in obese rabbits after treatment with a RD. Amine oxidase copper containing 3 (AOC3), phosphoglycerate dehydrogenase (PHGDH), and phosphoserine aminotransferase 1 (PSAT1) were upregulated in the glycine, serine, and threonine metabolism pathways, and extensive work revealed that these pathways are key players in the antibacterial ability [25,26]. AOC3 expressed by adipocytes catalyzes the oxidative deamination of amines to release H_2_O_2_, ammonia, and aldehyde [27]. With the finding about the importance of H_2_O_2_ in the insulin signaling pathway, a possible role of AOC3 in adipocytes may be found [28]. PHGDH and PSAT*1* are necessary enzymes participating in serine synthesis [29,30]. However, both PHGDH and PSAT*1* are regulators and associated with an increased risk for multiple cancer progression and metastasis, indicating a possible link between dieting and cancers [31,32]. Moreover, we also observed that some amino acid metabolism-related proteins were dysregulated, such as ubiquitin-specific peptidase 15 (USP15), ubiquitin family domain containing 1 (UBFD1), procollagen c-endopeptidase enhancer (PCOLCE), and alanyl aminopeptidase (ANPEP). Our results are consistent with the research on mental diseases in the rat model [10]. However, the specificity of these changes relative to other diseases requires further work to measure.

The metabolomics of obese rabbits treated with a RD showed changes of metabolites that were part of the phospholipid metabolism (phosphatidylcholine (PC), phosphatidylethanolamine (PE), diacylglycerol (DAG), phosphatidic acid (PA), and lysophosphatidic acid (LPA)) and fatty acid metabolism (arachidonic acid (AA)). Adipocytes function depends on the homeostasis of important cellular lipid mediators and lipid structural components of the biological membranes required for accurate functional responses [33]. After the HFD treatment, adipocytes initiate a series of membrane lipid remodeling in response to excessive energy intake. These changes in adipocytes may lead to a change in membrane composition, thereby resulting in changes in membrane fluidity and cellular physiology [34]. After RD treatment, we found that lower levels of most PC were expressed, but PE was increased in the RD group. The dynamic changes of PC and PE are associated with the structure and function of the membrane and could represent a compensatory response aimed at resisting the fluidity change [35,36]. Moreover, DAG, PA, LPA, and AA were decreased similarly in the RD group. These molecules are key contributors to membrane lipid remodeling and fluidity [33,37]. Interestingly, as an important molecule, PC is a major source of DAG, PA, LPA, and AA [38]. Various studies have pointed out the importance of DAG, PA, LPA, and AA for signal transmissions, which are pivotal second messengers controlling numerous cellular responses and can be further metabolized to other signaling molecules [39,40]. The data of proteomics also found that some proteins located in the membrane were significantly altered, which mainly included adhesion G protein-coupled receptor E5, aquaporin 4, frizzled class receptor 4, LDL receptor-related protein 1B, mannose receptor C type 2, notch receptor 2, Oryctolagus cuniculus very low-density lipoprotein receptor, annexin A3, annexin A5, and annexin A7. These proteins, except for annexin A3, annexin A5, and annexin A7, were downregulated, which suggests a deficit of signal transduction-related proteins in the RD group. Our study provided the possibility for a positive role of annexin in lipolysis. Increasing evidence revealed that the activities of annexin were facilitated through their diverse interactions with a plethora of lipids and proteins [41]. The annexin family are active in adipose tissue, and annexin A1, annexin A6, and annexin A3 have been linked to the control of adipocyte lipolysis and adiponectin release [41,42]. However, their functions in adipose tissue are less well-measured. Moreover, the downregulation of cytoskeleton-related proteins (cytoskeleton-associated protein 4, microtubule actin crosslinking factor 1, microtubule-associated scaffold protein 1, tubulin folding cofactor B, tubulin gamma complex-associated protein 3, and the fibronectin type III domain containing 3A) in the RD group suggested changes of the dynamics of the cytoskeleton [43]. The alterations of the cell shapes are accompanied by changes in the cytoskeletal organization and contacts with fibronectin, actin, and several cytoskeletal proteins [44]. Thus, we concluded that dieting results in membrane and cytoskeleton remodeling, along with a series of alterations in membrane composition and biophysical properties in the adipocytes.

Furthermore, a network analysis based on the pathway revealed that several identified nodes were notable, such as the reduction of MVA, AA, 15(S)-HpETE, farnesyl-diphosphate farnesyltransferase 1 (FDFT1), and sterol-C5-desaturase (SC5D) and enhancement of cholecalciferol, hydrocortisone, lipoxin B4, and lithocholic acid in the RD group. Given that these molecules strongly changed in multiple pathways, it is implied that they may play complex functions in regulating the metabolism or diseases impacted by dieting. It is expected that MVA was decreased because cholesterol synthesis has been associated with the mevalonic acid pathway [45]. Mevalonate metabolites affect not only transcriptional events but, also, post-transcriptional events that, in turn, impact various biological activities, including the energy metabolism [46]. The dysregulation of SC5D and FDFT1 may be responsible for the cholesterol-related molecular alterations, because they are the key roles involved in the processes of cholesterol synthesis and metabolism [47,48]. However, the discovery of decreased 15(S)-HpETE in the RD group was novel. 15(S)-HpETE is the product of AA formed in the 15-lipoxygenase pathway [49]. In adipose tissue, it is thought to have a causal relationship to antiangiogenics [50]. Extensive work revealed that inflammation and angiogenesis are interdependent [51]. Inflammatory cells participate in the angiogenic process by secreting proinflammatory and anti-inflammatory cytokines that can control endothelial cell proliferation, survival, and apoptosis, as well as their migration and activation, which are critical events in angiogenesis [52]. Due to the codependency of inflammation and angiogenesis, suppression of the angiogenic response may suppress inflammation [49]. Interestingly, cholecalciferol, hydrocortisone, lipoxin B4, and lithocholic acid were upregulated, which are known to counteract inflammation responses [53,54,55,56]. Moreover, a pathways analysis indicated that the above molecules have effects on many pathways, including sphingosine and sphingosine-1-phosphate metabolism, the metabolism of lipids, the short of Lipoxin, ovarian steroidogenesis, etc. In addition to the above molecular changes, the levels of branched fatty acid esters of hydroxy fatty acids (FAHFA) were markedly increased. Our work supports the early study that lipolysis in adipocytes is associated with marked increases in the FAHFA levels [57]. PAHFA is a novel class of endogenous lipids that also shows a great impact on lipid inflammation in the obese population [58]. Thus, we speculated that dieting regulated the expression of anti-inflammatory factors, thereby mitigating the inflammation induced by HFD. However, this regulatory mechanism has not been fully studied.

Taken together, we applied an integrated proteomics and metabolomics analysis to investigate the effects of dieting on the proteins and metabolites in obese rabbits. We found that metabolomics and proteomics were changed markedly after rabbits treated with a RD, and the amino acid metabolism, lipid metabolism, and membrane and cytoskeleton reconstruction may be the key processes during dieting. Moreover, the body may change the expression of anti-inflammatory factors to fight inflammation induced by HFD. Our study provides further insights into the research of dieting and may contribute to the prevention and treatment of obesity. However, multiple limitations exist in this study. For example, given that age influences the metabolic rates associated with proteins and metabolites metabolism, the rabbit age is a critical factor that should be taken seriously. Since the specimens were animals, the number of specimens was relatively small. Moreover, we did not use a different method or database to verify the analysis results. One method cannot cover every aspect of the real physical changes. Thus, the functional verification of these DE proteins and DE metabolites and their network will be important to consider in the future.

## Figures and Tables

**Table 1:** Adipocytes area, diameter, number, and density in high-fat diet (HFD) and restricted diet (RD) rabbits.

Parameters	HFD	RD
Adipocytes area (μm^2^)	1098 ± 92.13	793.6 ± 57.55 *
Adipocytes diameter (μm)	39.81 ± 1.485	34.44 ± 1.038 **
Adipocytes number	37.22 ± 4.651	54.89 ± 3.3448 **
Adipocytes density (number/mm^2^)	417.8 ± 52.32	619.4 ± 37.73 **

Data are shown as the mean ± SEM (*n* = 9). Three HFD and three RD rabbits were used for sampling, and three duplicate samples were collected from each rabbit. A *t*-test was used for the differences between the HFD and RD rabbits. * *p* < 0.05 and ** *p* < 0.01.

## Data Availability

All data generated or analyzed during this study are included.

## Supplementary Materials

The following are available online at https://www.mdpi.com/article/10.3390/biology10040321/s1: Table S1: The list of all identified proteins. Table S2: The list of DE proteins between the HFD and RD groups. Table S3: The GO analysis results of the DE proteins. Table S4: The list of DE metabolites between the HFD and RD groups. Table S5: The annotation results when the DE metabolites were compared with the HMDB database. Table S6: The annotation results when the DE metabolites were compared with the LIPID MAPS database. Table S7: Integrated pathway analysis of the proteomics and metabolomics.
